# Predictors of perinatal mortality in rural population of Northwest Ethiopia: a prospective longitudinal study

**DOI:** 10.1186/1471-2458-13-168

**Published:** 2013-02-23

**Authors:** Gashaw Andargie, Yemane Berhane, Alemayehu Worku, Yigzaw Kebede

**Affiliations:** 1 The University of Gondar, College of Medicine and Health Sciences, Institute of Public Health, P.O. Box 196, Gondar, Ethiopia; 2 Addis Continental Institute of Public Health, Addis Ababa, Ethiopia; 3 School of Public Health, Addis Ababa University, Addis Ababa, Ethiopia

**Keywords:** Perinatal mortality, Early neonatal mortality, Still birth, Ethiopia

## Abstract

**Background:**

Perinatal mortality is one of the serious challenges in meeting maternal and child Millennium Development Goals in developing countries. Identifying its predictors is an important step to develop focused and appropriate health interventions for reducing perinatal deaths. This study therefore aims at identifying predictors of perinatal mortality in a rural setting in northwest Ethiopia.

**Methods:**

A prospective longitudinal study was conducted at Dabat Health and Demographic Surveillance site, northwest Ethiopia, from November 2009 to August 2011. Data were collected by interviewing the mothers or guardians of eligible children. Multiple logistic regressions were employed to identify potential predictors.

**Results:**

A total of 1752 eligible children were included in the study. Perinatal mortality rate in the study population was 50.22 per 1000 (95% CI: 39.99, 60.46) total births. In multiple logistic analysis, previous still birth [(AOR = 8.38, 95% CI: 3.94, 17.83)], twin birth [(AOR = 7.09, 95% CI: (3.22, 15.61)], not receiving tetanus toxoid vaccine during the index pregnancy [(AOR = 3.62, 95% CI: 1.57, 8.34)], short birth interval of less than 24 months [(AOR = 2.58, 95% CI: (1.61, 4.13)], maternal illiteracy [(AOR = 4.83, 95% CI: (1.45, 16.05)] and mothers’ running own business [(AOR = 5.40, 95% CI: 1.40, 27.96)] were the main predictors associated with increased risk of perinatal death.

**Conclusions:**

Predictors of perinatal death in the study area are easily recognizable and potentially preventable with the existing maternal health programs. Efforts need to be intensified in expanding maternal and newborn health services to significantly reduce perinatal mortality in rural settings.

## Background

Perinatal Mortality Rate (PNMR) is known to be one of the key health status and socio-economic indicators of the community [1]. It is specifically a sensitive index of the quality of prenatal, obstetric, and early neonatal care available to women and newborns in any setting [2,3]. There are an estimated ten perinatal deaths for each maternal death [4]. At least 75% of the perinatal deaths that occur in developing countries are caused by problems that also kill women. These include prolonged/obstructed labour, puerperal sepsis, eclampsia, women’s nutrition, infection and hemorrhage [5].

Global estimates show that the PNMR in developed regions of the world is about 10 per 1000 total births compared to 50 per 1000 total births in less developed regions [1]. Sub-Saharan Africa has the highest rate of perinatal deaths, 56 per 1000 births [1]. According to the Ethiopian Demographic and Health Survey (EDHS) 2011, PNMR in Ethiopia was estimated at 46 per 1000 births [6], however, the rate varies by regions ranging from 20 to 58 deaths per 1000 births. The PNMR was 55 per 1000 births in the Amhara Regional State, where this study was conducted [6].

Although the country has intensified efforts to provide basic maternal and child health services through the Health Extension Program [7,8], reducing perinatal deaths remains a formidable challenge to achieving Millennium Development Goal 4 in rural Ethiopia [9,10], where only 4.1% of deliveries are attended by skilled birth attendants [6].

The causes of perinatal morality are multiple and include previous still births [11], twin births [12,13], being unimmunized for two doses of tetanus during pregnancy [14], birth interval of less than 24 months for the index child [15-17], illiteracy [18-20] mother’s being farmer by occupation [21].

In a country like Ethiopia, where only 10% of the births are delivered at a health facility, documenting the rate and causes of perinatal mortality through a community based prospective study is an important input for planning effective interventions. Previous estimates in Ethiopia are either largely hospital-based [22,23] or retrospective household surveys [6] which have obvious limitations associated with recall and selection bias. In addition, the risk factors for the perinatal mortality are poorly documented and understood in northwest Ethiopia. Thus, the objective of this prospective follow up community-based study was to estimate PNMR and to identify the potential risk factors in rural communities of northwest Ethiopia.

## Methods

### Study setting

**Figure 1:**
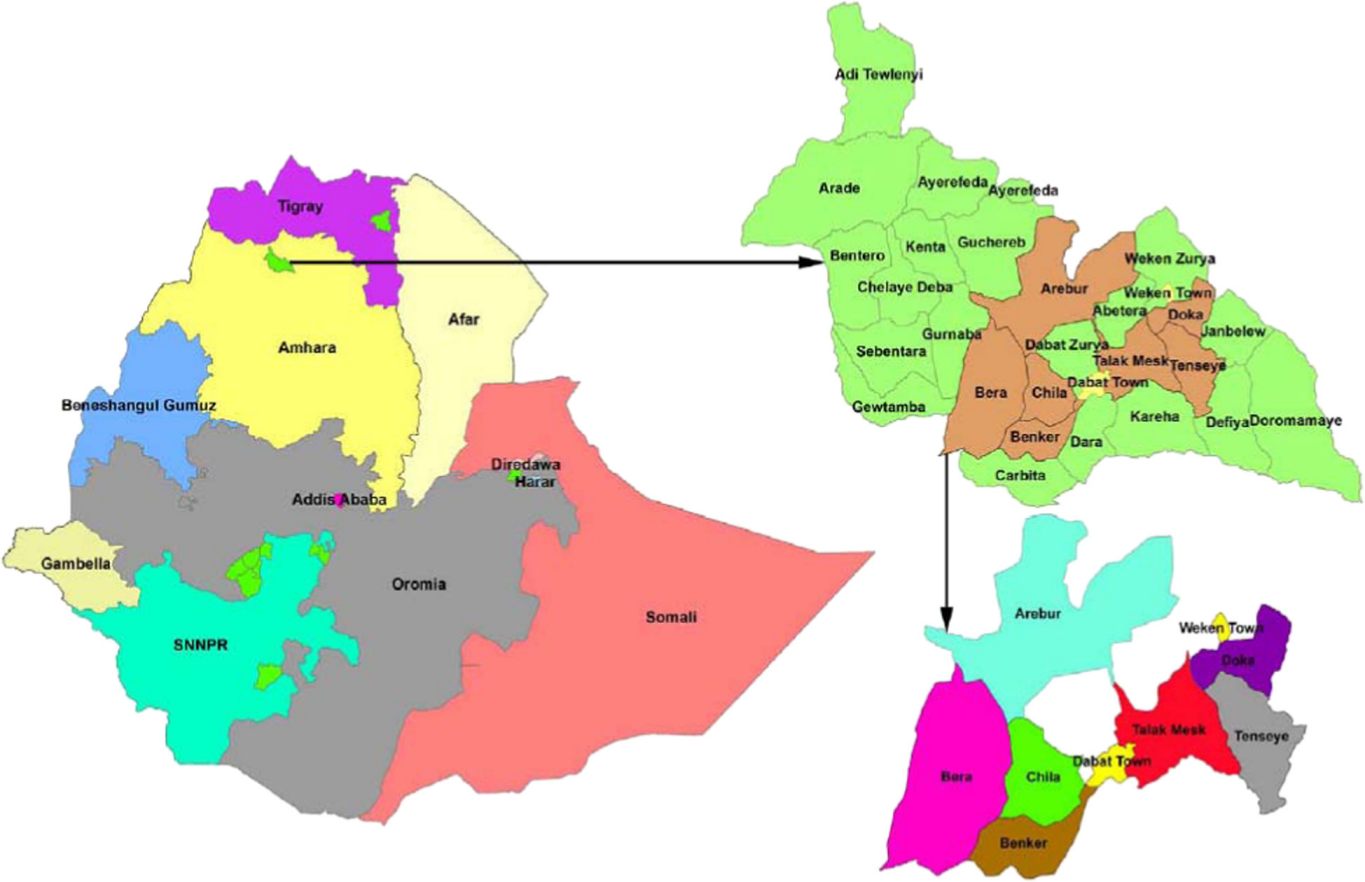
**Map of Dabat Health Demographic Surveillance Site at Dabat district in northwest Ethiopia.** Areas shaded with different colors show the different *Kebeles* in the Dabat HDSS.

The study was conducted at the Dabat Health and Demographic Surveillance Site (HDSS) located at Dabat district in northwest Ethiopia (Figure 1). Dabat has one district hospital, two health centers, three health stations, and twenty-nine health posts that are providing health services to the community [24]. The HDSS covers ten (three urban and seven rural) randomly selected *kebeles* (the lowest local administrations in the Ethiopian context). At the time of the study, there were 46,165 people in the DHSS with 49% of children under 14 years. The HDSS has been collecting information on vital events like, birth, death, migration, and pregnancy registrations and outcomes thereof on quarterly and regular bases since 1996 [24]. 

### Study design

The study was part of a larger prospective follow-up research on infant mortality. The perinatal deaths (stillbirths and early neonatal deaths) which occurred during the follow-up period from November 2009 to August 2011 were included.

### Study population and sample size

All mothers living in the site and reported in second/third trimester of pregnancy were invited to join the prospective longitudinal study. Pregnancy was detected through interview by trained data collectors. For identifying the predictors of perinatal mortality, all deaths during the perinatal period were compared to those who survived the perinatal period.

### Data collection

Data were collected using a structured questionnaire adapted from the UNICEF multiple indicator cluster survey questionnaire [25]. The questionnaire was pre-tested on an adjacent population. The data collection was overseen by three field supervisors who had previous experiences in conducting similar studies in the study area. Seventeen data collectors with high school education and previous experience were recruited for the study. Prior to the actual data collection, a five day intensive training was given to data collectors and field supervisors about data collection tools and study procedures. The data collectors and field supervisors were assisted in the field by local informants who were residents of the study villages. The local informants were trained to report the end of pregnancy to the data collectors as soon as they identify the event irrespective of the pregnancy outcome. Data quality was maintained through regular on-site supervision and by conducting random rechecking of 5% of the respondents in each locality.

### Study variables

Perinatal mortality was the outcome variable. It was a dichotomous variable indicating whether the child is alive or dead through the perinatal period. The explanatory variables included as potential predictors for perinatal death were maternal age at pregnancy outcome, maternal and paternal educational status, maternal and paternal occupation, marital status of parents, taking of at least two prophylactic dose of TT vaccine during the index pregnancy, birth interval (less or more than 24 months) for the index child, maternal previous still births, ever use of family planning methods, sex of the neonate and whether the neonate was single or multiple birth.

### Data management and analysis

Double data entry was done on a regular basis using EpiInfo for windows Version 3.5. The error rate was below 5%, except for spelling errors for alphanumeric variables. The data were transferred from EpiInfo to STATA Version 11 software and cleaned by reviewing frequency tables, logical errors, and checking outliers. Analysis was done in a series of steps.

A bivariate analysis was carried out to examine the relationship between perinatal death and the potential predictors without adjusting for other covariates. Then all potential predictors with P-value less than 0.20 in the univariate analysis were used to include more potential predictors. Factors previously reported to be associated with perinatal deaths were entered into a multiple logistic regression model to examine their effects simultaneously.

In the multivariate analysis model, possible associated factors were examined for evidence of collinearity which was reflected either by the changes in the direction of effect between the univariate and multivariate analysis or implausible standard errors for a particular variable. The final model used was found to be a valid model with Hosmer-Lemeshow goodness of fit with an associated P value greater than 0.05. Odds ratios (ORs) and 95% confidence intervals (CIs) were computed using logistic regression models to assess the relationship between perinatal mortality and each selected variable.

### Ethical consideration

Ethical approval was obtained from the University of Gondar Ethical Review Board. A formal letter was written to the local district administrative and health offices. Informed verbal consent was secured from all mothers or guardians of the study participants. The right of the respondent to withdraw from the interview or not to participate was informed and respected. The final results of the research will be communicated to government offices and most importantly to the study subjects and members of the community through health extension workers.

## Results

The study comprised 1752 pregnant women followed from their second/third trimester through 7^th^ day post delivery (Table 1). A total of 88 perinatal deaths were identified at the end of the follow up period. The PNMR in the study was 50.22 per 1000 total births (95% CI 39.99, 60.5). The contribution to this PNMR from stillbirths was 23.4 per 1000 births (95% CI: 16.31, 30.48) and that of early neonatal deaths was 27.5 per 1,000 live births (95% CI 19.25, 34.39).

**Table 1:** **Number of births, perinatal deaths and mortality rates in rural population of northwest Ethiopia, 2009–201**1

**Description**	**Total**	**(95% Confidence Interval)**
Total births	1752	
Total live births	1711	
Still births	41	
Early neonatal deaths	47	
Perinatal deaths	88	
Still birth rate (per 1000 birth)	23.4	(16.3,30.5)
Early neonatal death rate (per 1000 live births)	27.47	(19.3,34.4)
Perinatal mortality rate (per 1000 births)	50.23	(39.9, 60.5)

In the multivariate logistic regression, the following factors were associated with increased risk of perinatal mortality. Among the socio-demographic factors, maternal illiteracy [AOR = 4.83, 95% CI (1.45, 16.05)], and working on own business [AOR = 5.40, CI (1.40, 27.96)]; among child factors being a female child [AOR = 1.61, 95% CI (1.04, 2.67)] and multiple/twin birth [AOR = 7.09, 95% CI, (3.22, 15.61] were significantly associated with perinatal death.

Maternal factors associated with perinatal mortality were history of stillbirth [AOR = 8.38, CI (3.94, 17.83)], birth interval of less than two years [AOR = 2.58, CI (1.61, 4.13)], and not immunized mothers for at least two doses of TT vaccine during pregnancy [AOR = 4.12, CI (1.77, 9.59)] (Table 2).

**Table 2:** Association between selected predictor factors for perinatal mortality in rural population of northwest Ethiopia, 2009–2011

**Descriptions**	**Perinatal death**		**COR (95% Confidence Interval)**	**AOR (95% Confidence Interval)**
	**Yes: # (%**)	**No: #(%)**		
**Marital status of mothers**				
Married	77 (4.7)	1547 (95.3)	0.53 (0.27,1.02)	0.47 (0.21,1.08)
Single/widowed/separated	11 (8.6)	117 (90.7)	1	1
**Educational status of mothers**				
Illiterate	78 (6.1)	1192 (93.9)	3.94 (1.43,10.87)	4.83 (1.45,16.05)
Primary/informal	6 (2.5)	231 (97.5)	1.57 (0.44,5.62)	1.75 (0.43,7.25)
Secondary & higher	4 (1.6)	245 (98.4)	1	1
**Fathers educational status**				
Illiterate	57 (5.6)	966 (94.4)	2.2 (0.92,5.08)	2.69 (0.78,9.34)
primary school/informal	25 (5.0)	478 (95.0)	1.92 (0.78,4.74)	2.44 (0.71,8.40)
Secondary/higher	6 (2.7)	220 (97.3)	1	1
**Mothers occupation**				
Farmer	3 (12.0)	22 (88.0)	7.23 (1.37,38.06)	5.02 (0.79,31.71)
Own business	5 (6.2)	76 (93.8)	3.49 (0.81,14.97)	5.40 (1.40,27.96)
Housewives	77 (5.2)	1403 (94.8)	2.90 (0.91,9.30)	2.73 (0.75,9.94)
Others	3 (1.9)	159 (98.1)	1	1
**Fathers occupation**				
Student	6 (10.5)	51 (89.5)	2.97 (0.80,10.99)	1.04 (0.19, 5.62)
Farmer	72 (4.9)	1402 (95.1)	1.32 (1.47,3.69)	0.37I (0.08,1.64)
Own business	6 (5.3)	107 (94.7)	1.44 (0.40,5.27)	1.17 (0.25,5.47)
Other	4 (3.7)	103 (95.9)	1	1
**Sex of infants**				
Male	55 (6.2)	835 (93.8)	1.67 (1.06,2.58)	1.61 (1.04,2.67)
Female	33 (3.8)	828 (96.2)	1	1
**Type of birth out comes**				
Multiple	12 (21.1)	45 (78.9)	5.7 (2.89,11.18)	7.09 (3.22,15.61)
Single	76 (4.5)	1619 (95.5)	1	1
**Birth interval for the index child**				
< 24 months	40 (7.6)	483 (92.4)	2.04 (1.32,3.14)	2.58 (1.61,4.13)
> = 24 months	48 (3.9)	1181 (96.1)	1	1
**History of Abortion**				
Yes	8 (9.3)	78 (90.7)	2.03 (0.95,4.35)	1.31 (0.54,3.15)
No	80 (4.8)	1586 (95.2)	1	1
**Previous still births**				
Yes	15 (27.8)	39 (70.9)	8.6 (4.52,16.24)	8.38 (3.94,17.83)
No	73 (4.3)	1625 (95.8)	1	1
**Ever use of family planning Methods**				
Yes	19 (4.4)	415 (95.6)	0.83 (0.49,1.4)	1.09 (0.58,2.03)
No	69 (5.2)	1249 (94.8)	1	1
**Maternal TT vaccine**				
No	81 (6.0)	1263 (94)	3.67 (1.68,8.02)	3.62 (1.57, 8.34)
Yes	7 (1.7)	401 (98.3)	1	1

## Discussion

The PNMR among the rural population of northwest Ethiopia was very high, and the finding is in agreement with previous national reports by WHO, 2007 [1], and EDHS, 2011 for the Amhara Regional State of Ethiopia [6]. A recent community-level study conducted in Burkina Faso reported a PNMR of 79.0 per 1000 total births which was greater than the findings of this study [13]. The possible reason for the difference might be the fact that the Ethiopian Government has initiated a community based health service package (Health Extension Package) that intensified the availability of maternal and child health services through the Health Extension Program [7]. The health extension workers provide family planning and immunization services; they promote preparedness for birth and readiness for complications, and active management of the third stage of labor among others [8].

The risk factors identified in this study, namely low educational status of mothers, maternal ownership of business as occupation, history of still birth, short birth interval of less than two years, mother’s being unimmunized for at least two doses of TT Vaccine during pregnancy are in agreement with findings of previous studies done in other developing and middle income countries [11,18,21].

In this study, the highest perinatal mortality was observed among illiterate mothers, and the finding is similar to those of other studies in developing countries [19,20]. Evidences from other developing countries also indicated that increased levels of mother’s education were observed to be associated with improved chances of infant survival [26]. Our findings emphasize the need for encouraging female literacy which by itself is expected to provide multiple benefits and better chances for alleviating poverty and poverty-related health problems [26]. Education can improve economic status, access to health care, and birth spacing which are known to reduce the risk of perinatal mortality [27].

The results of this study also specified that twin are more likely to die during the perinatal period compared to children born singletons, as has been reported by a similar study in rural Burkina Faso [13]. Intrauterine growth restrictions and birth defects and/or disabilities that are common in multiple pregnancy increase vulnerability to perinatal death [12,28]. These multiple pregnancies require special and expensive medical care [28] which is not accessible and available in our study area.

In our study, women who had experienced previous still birth had an increased risk of losing their children in the consecutive perinatal period, and this finding is consistent with findings of a previous study [29]. Moreover, genetic and environmental factors can lead to repeated occurrences of small-for-gestational age [SGA], birth/intrauterine growth restriction, preeclampsia, and placental abruption which can eventually cause untreated perinatal death [29,30].

A short birth spacing of less than 24 months was associated with an increased risk of perinatal mortality because of the well known phenomena related to sibling competitions recognized as the maternal depletion syndrome [15-17]. The syndrome is also associated with premature rupture of membranes and puerperal endometritis [31,32] which can cause perinatal deaths. As reported by previous studies, mothers who received at least two doses of maternal tetanus toxoid vaccinations could significantly reduce perinatal mortality [14,33].

Even though every effort was made to maintain the quality of the data, the study has limitations that should be noted when interpreting the results. First of all, readers should be cautious since the study findings show a wide confidence interval because of the small sample size of the deaths. Secondly, although the study design was prospective, it was not able to measure birth weight of the neonates and other clinical conditions which are important predicators of perinatal mortality because most deliveries took place at home and the researchers could not manage to secure resources to do anthropometric and clinical assessments immediately after birth.

## Conclusion

In summary, perinatal death in the study area was largely due to preventable conditions for which existing maternal health programs have been proven interventions. Therefore, urgent actions are required to expand the existing maternal and newborn health services in the short term to rapidly and significantly reduce perinatal mortality in rural settings of Ethiopia as the country is making progress to achieving its long term vision of making poverty history.

## Competing interests

The authors declare that they have no competing interests.

## Authors’ contributions

GA, YB, AW and YK participated in all steps of the study from its inception to the write up. All authors have reviewed and approved the submission of the manuscript.

## Authors’ information

GA is a Public Health expert specializing on child health research at the University of Gondar. He is an active member of the Dabat Demographic Surveillance and Health research team.

YB is a senior professor of epidemiology and public health at Addis Continental Institute of Public Health. He has extensively published on maternal and child health issues.

AW is an Associate Professor at the Department of Epidemiology and Biostatistics of Addis Ababa University, Ethiopia. He has been teaching Biostatistics and research Methods for many years and has participated in many field/clinical trials.

YK is a senior professor of public health at the Institute of Public Health, the University of Gondar. He has been teaching several courses in public health, epidemiology, and research methods in Ethiopian universities for more than ten years. He has collaborated with national and international colleagues in different research projects. He has advised doctoral and MPH students in Ethiopia.

## Pre-publication history

The pre-publication history for this paper can be accessed here:

http://www.biomedcentral.com/1471-2458/13/168/prepub
